# Treatment and Diagnostic Approach for Lhermitte-Duclos Disease and Suspected Cowden Syndrome

**DOI:** 10.7759/cureus.62968

**Published:** 2024-06-23

**Authors:** Ricardo García-Iturbide, Joel A. Velázquez, Isauro Lozano Guzmán, Jesus E Falcon-Molina, Marco A Rodríguez, Adrian Sánchez-Gómez, Jesùs R Heras Lorenzana, Eric M Estrada Estrada

**Affiliations:** 1 Neurological Surgery, Hospital de Especialidades Centro Médico Nacional Siglo XXI, Instituto Mexicano del Seguro Social (IMSS), Mexico City, MEX; 2 Pathology, Hospital de Especialidades Centro Médico Nacional Siglo XXI, Instituto Mexicano del Seguro Social (IMSS), Mexico City, MEX; 3 Neurological Surgery, Hospital Regional 1ro de Octubre, Instituto de Seguridad y Servicios Sociales de los Trabajadores del Estado (ISSSTE), Mexico City, MEX; 4 Neurological Surgery, Hospital Regional de Alta Especialidad Bicentenario de la Independencia, Instituto de Seguridad y Servicios Sociales de los Trabajadores del Estado (ISSSTE), Tultitlán de Mariano Escobedo, MEX

**Keywords:** tiger-striped appearance, pten syndrome, cowden syndrome, lhermitte-duclos disease, dysplastic cerebellar gangliocytoma

## Abstract

Lhermitte-Duclos disease (LDD) is a rare entity, which may or may not be associated with Cowden syndrome (CS). The authors present a 26-year-old male with a history of emergency treatment due to acute obstructive hydrocephalus and apparent Chiari malformation. In posterior evaluation, mild cerebellar symptoms, mucocutaneous lesions, and a left hemispheric cerebellar lesion were evident.

Initially, with the clinical evidence and the radiological study report of a cerebellar tiger-striped lesion, LDD with associated CS was suspected, and a genetic protocol was performed. The protocol included an endoscopy and thyroid ultrasound, and with symptom progression, a new neurosurgical procedure was performed. To complete the approach, we used the clinical criteria for PTEN hamartoma tumor syndrome established in 2013, and CS was diagnosed in the patient.

In patients with radiological and clinical suspicion of LDD and CS, it should be mandatory to investigate the presence of other types of tumors due to their association with PTEN hamartomatous tumor syndrome, and in the absence of genetic study, the clinical criteria previously established in the literature should be sufficient to establish the diagnosis.

## Introduction

Cerebellar dysplastic gangliocytoma (CDG) or Lhermitte-Duclos disease (LDD) is a cerebellar lesion characterized by dysplastic ganglion cells that adapt to the cortical architecture and thicken the cerebellar folia. The World Health Organization (WHO) in the 2021 version of the Classification of Tumors of the Central Nervous System determined LDD as a "glioneuronal and neuronal tumor" Grade 1 [1]. Currently, there is still controversy about considering this lesion as a dysplasia, neoplasia, hamartoma, or some combination of these [2,3]; however, some authors are emphatic in considering it a hamartoma [4,5].

LDD is considered a rare disease, with fewer than 300 cases reported since its initial description in 1920 by Lhermitte and Duclos. The age of presentation ranges from three to 75 years, with the largest number of cases between the 3rd and 6th decade of life and without apparent gender preference [6-8]. Therefore, there are not enough studies to establish its specific incidence and prevalence.

LDD belongs to a group of diseases known as "PTEN hamartomatous tumor syndromes," along with Cowden syndrome (CS), Bannayan-Riley-Ruvalcaba syndrome, Proteus syndrome, and autism-macrocephaly syndrome [9,10]. This entity is present in up to 30% of patients with CS, forming part of the major criteria for its diagnosis [10,11].

## Case presentation

**Figure 1:**
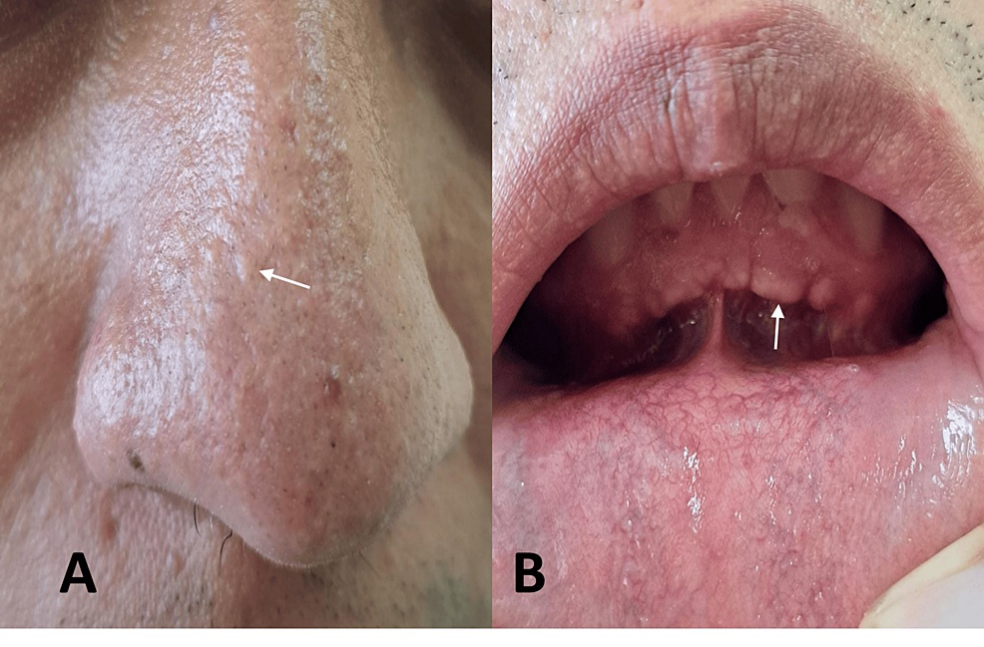
Skin lesions. (A) Multiple skin-colored flat-topped papules in the nasal dorsum (arrow) and (B) multiple gingival whitish surfaced papillomatous lesions with a cobblestone appearance (arrow).

A 26-year-old male had a two-year history of progressive intermittent headache, followed by nausea, vomiting, incoordination, and left-lateralized gait disturbance. Due to his symptoms, he was taken to a regional hospital where acute hydrocephalus was reported and a ventriculoperitoneal shunt was performed. After the procedure, he was referred to our hospital with a report of Chiari malformation by computed tomography (CT). During our first consultation, the patient reported improvement in his symptoms and a history of resection of lipomas on his back and left leg. The physical and neurological examination revealed a head circumference of 60 cm, papillomatous nasal lesions (Figure 1A), gingival papules (Figure 1B), cerebellar ataxia with left dysmetria, and mild dysdiadochokinesia.

**Figure 2:**
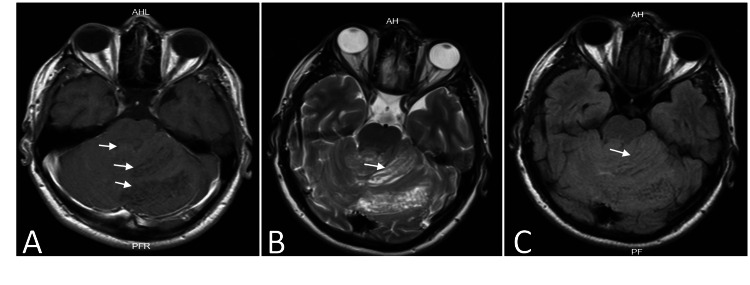
Preoperative MRI. (A) T1-weighted imaging (T1WI) with gadolinium showing an iso- and hypointense left cerebellar hemisphere and vermian lesion without contrast enhancement (arrows) compressing the fourth ventricle. (B) T2-weighted imaging (T2WI) of the lesion with alternating iso- and hyperintense bands related to a tiger-striped sign (white arrow). (C) In fluid-attenuated inversion recovery (FLAIR), the bands are seen as iso and hypointense (white arrow).

Magnetic resonance imaging (MRI) of the brain showed a diffuse lesion in the left cerebellar hemisphere and vermis with poorly defined borders causing cerebellar folia enlargement and fourth ventricular compression with tonsillar herniation. The lesion was iso and hypointense on T1-weighted imaging (T1WI) and showed no gadolinium enhancement (Figure 2A). T2-weighted imaging (T2WI) showed a “tiger-striped appearance” (Figure 2B), and on fluid-attenuated inversion recovery (FLAIR), the stripes were hypo and isointense (Figure 2C). Due to imaging and clinical findings, LDD in association with CS was suspected. The patient was referred to the genetic department, where a thyroid ultrasound and panendoscopy reported a thyroid nodule of Thyroid Imaging Reporting and Data Systems (TIRADS) 3 and gastric and colonic polyps.

The patient was referred to our service with an increase in cerebellar symptoms, so we performed a left lateral suboccipital craniotomy with resection guided by neuronavigation in the Concorde position and with the head fixed at three points in the Mayfield clamp. The surgical view revealed enlarged and widened cerebellar folia, a palish and friable lesion. Hemostasis was secured, and the surgical specimen was sent to the department of pathology for definitive study. The patient was discharged three days after the procedure with a slight improvement in cerebellar symptoms.

**Figure 3:**
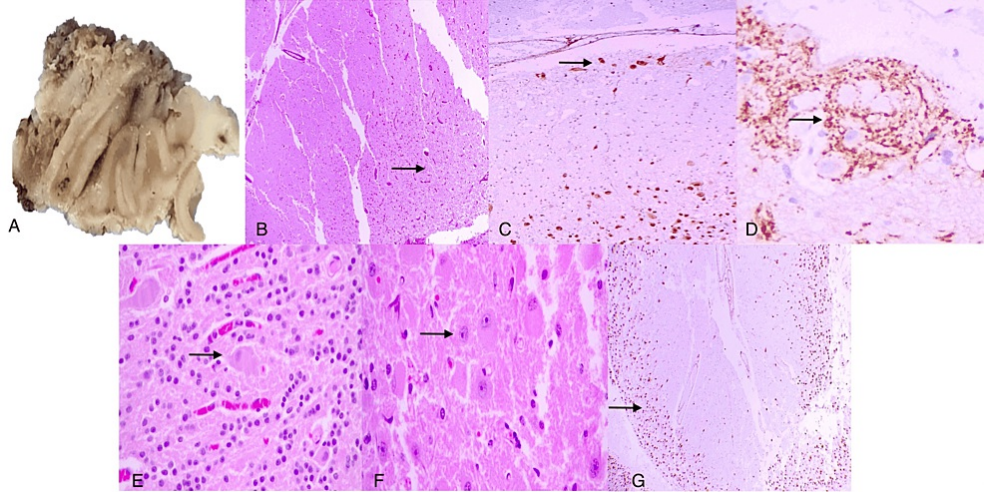
Histopathology. (A) Macroscopic view of the cerebellar lesion showing thickened folia of variable sizes, which show a light brown rim on the periphery and a dark brown center. (B) Hematoxylin & eosin (HE) (4x) showing the absence of granular layer and Purkinje cells with preservation of molecular layer (arrow). (C) NeuN immunohistochemistry (4x) showing few subpial dysplastic neurons (arrow). (D) Synaptophysin immunohistochemistry (40x) showing increased density of neurosecretory granules (arrow) in neuronal subpial foci. (E) HE (40x) showing dysplastic neurons (arrow) immersed in the residual granular layer. (F) HE (40x) showing dysplastic neurons (arrow) with a background similar to a neuropil with complete replacement of the granular layer. (G) NeuN immunohistochemistry (4x) showing dysplastic neurons replacing the granular layer (arrow) and preservation of the molecular layer.

The surgical specimen was reported as a pale grayish soft tumor with irregular thickening of the left cerebellar folia (Figure 3A). The histopathology report described a replacement of the granular layer by dysplastic ganglion cells, attenuated Purkinje cell layer, and subpial neurons in the molecular layer confirmed with immunohistochemistry positive for NeuN and synaptophysin, which are characteristic findings of cerebellar dysplastic gangliocytoma or LDD (Figures 3B-3G).

With the confirmed diagnosis by histopathology of LDD and the clinical findings (macrocephaly and skin lesions), the patient was diagnosed with CS in the PTEN hamartomatous tumor syndromes group, accomplishing three major clinical criteria for diagnosis. The patient is still undergoing further investigations for the other manifestations reported (thyroid nodule and gastric and colonic polyps). The molecular study could not be performed in our hospital; however, we used the Cleveland Clinic PTEN Risk Calculator (https://www.lerner.ccf.org/genomic-medicine/ccscore/), obtaining a score of 27, corresponding to a mutation probability of 72% in the PTEN gene.

## Discussion

LLD is a rare disease and there is no incidence reported because of the few cases in the literature. Typically, it occurs with mutations in the PTEN germinal line in 85% of cases in adulthood, while in the pediatric population, it presents with other genetic manifestations, suggesting different biological mechanisms between these groups [12]. There is still controversy about the origin, suggesting the cerebellar granular neuron is the primary origin cell with an aberrant combination and hypertrophy of granular cells [13-15].

The clinical manifestations of LDD are related to the mass effect generated in the posterior fossa and the cerebellar edema, which may produce hydrocephalus with related intracranial hypertension, cerebellar signs (ataxia, dysmetria, dysdiadochokinesia, and dizziness), and headache of variable intensity, which may be enhanced with the Valsalva maneuver; however, there are cases reported as asymptomatic [2,4,7].

The diagnosis of LDD is typically based on radiology and histopathological findings. In radiology studies, the cerebellar architecture is altered, showing the thickness of the cerebellar folia. Some cases present as a cystic lesion with or without fourth ventricle displacement [1,15]. CT scans show areas of iso- and hypodensity at the lesion site, but frequently, a type 1 Chiari malformation due to tonsillar herniation is reported as the only finding [4,6,15]. MRI is the diagnostic study of choice. On T1WI, it shows iso- and hypointense lesions with stripes, and in contrast, it can show the perilesional vasculature with or without minimal enhancement of the lesion. On T2WI, the white matter is hyperintense with hypo- or isointense gray matter, which is called the classic “tiger stripe sign,” also described as a tigroid appearance (Figure 2), with similar findings in the FLAIR sequence [15-20].

The macroscopic appearance is described as a red palish lesion with hemorrhagic and cystic areas, with hypertrophic cerebellar tissue in a circular patron with deep extension [1,2]. The characteristic histopathological findings of LDD are a relative conservation of the cerebellar architecture with folia thickness as a consequence of a diffuse granular and molecular layer, which usually contains ganglion cells of multiple sizes, morphologically dispersed cells, and an attenuated or absent Purkinje cell layer. In the present case, abundant ganglion cells were reported in the granular layer, in which the Purkinje cells were partially conserved, and there was a conserved molecular layer with small groups of subpial ganglion cells (Figures 3B-3G).

The abnormally myelinated axons in the molecular layer usually present immunoreactivity to synaptophysin, NeuN, neurofilaments, and mTOR, with a characteristic loss of PTEN expression. They can be positive for Olig2 and GFAP, negative for TP53, S-100, and IDH1 R132H, and have minimal Ki-67, usually <1% [1,13-15,21].

These histopathologic findings show the variable spectrum of morphological changes that can be observed in the same lesion. It is controversial whether this lesion is a dysplasia, neoplasia, or hamartoma; in our case, we consider this lesion to be a dysplasia, because of the substitution of granular cells for dysplastic ganglion cells, which may be altered neuronal dedifferentiation in the granular layer, as proven by small spots of dysplastic ganglion cells in the conserved granular layer with alternated zones of complete substitution. In our case, the molecular layer was conserved with small groups of dysplastic ganglion cells, which suggests a migration and maturation etiology.

Two types of treatment can be established for these injuries: (a) conservative management offered to patients with small lesions that cause no or minimal symptoms, with serial follow-up through imaging studies; and (b) surgical treatment when the symptoms limit the patient's quality of life or condition other alterations, such as tonsillar herniation. Complete resection of the lesion is considered the gold standard; however, due to the possible extension of the lesion toward other structures, sometimes only a partial resection can be performed [2,5,7]. In cases where hydrocephalus is conditioned, a ventricular shunt will be placed depending on the patient's clinical context. There are reported cases of lesion recurrence (associated with partial resections), whose treatment is limited to a new surgical resection, with excellent results [2,8]. Due to the characteristics previously described, there is no need for other types of treatment [1,2].

In addition, the association between LDD and CS should be considered since both are linked to germline mutations in the PTEN gene on chromosome 10. However, despite having been previously reported, this relationship is not precise, as patients with LDD may or may not generate the clinical and systemic features of CS.

CS is an autosomal dominant hereditary syndrome that determines a predisposition to cancer, characterized by macrocephaly, mucocutaneous abnormalities, and benign and malignant tumors. It is a rare entity, with an approximate incidence of 1/200,000 people, formally described as a specific entity in 1963 by Lloyd and Dennis in a family with the surname Cowden. The average age of presentation is 40 years, with reported cases from 10 to 65 years. Clinically, this entity presents involvement at the level of multiple apparatuses and systems [10,21,22].

The skin is the organ most frequently affected in CS, presenting as trichilemmomas and inverted keratosis follicularis in facial or oral locations. Other associated lesions are lipomas, hemangiomas, and lymphangiomas. There are mucosal lesions, described as smooth-surfaced papules, gingival papules, or involuted warts, and even the presence of a scrotal tongue [10,23,24]. Thyroid involvement occurs in just over half of the patients, with findings ranging from thyroiditis, goiter, thyroid nodules, or carcinoma. The breast is another affected organ, representing up to 84% in some series, with fibrocystic disease or infiltrating ductal carcinoma as the two predominant lesions found. At the gastrointestinal level, polyposis is the most frequently identified pathology in the colon or stomach; hamartomatous polyps are the most frequently associated subtype; and another gastrointestinal manifestation of CS is glycogenic acanthosis in the esophagus. At the genitourinary level, women are almost exclusively affected: endometrial cancer and uterine leiomyomas are the most frequently associated manifestations, as well as renal cell carcinoma; other disorders are hypospadias, urinary tract malformations, menstrual disorders, polycystic ovary syndrome, and spontaneous abortions or fetal death. In the respiratory system, pulmonary cysts have been documented in 80-100% of cases in patients with PTEN mutations, with solid nodules in up to 87%. Finally, with respect to the nervous system, cerebellar dysplastic gangliocytoma is the most frequently associated entity, with a prevalence of 15-32% associated with CS; retardation in psychomotor development can be present in 12 to 20% of cases; in patients with PTEN alteration, approximately 50% of cases have been associated with motor or language delay but with normal intelligence in adulthood [10,25].

Once patients with both CS and LDD are identified, they should be constantly monitored because of the five to seven times higher risk of developing tumors compared to the general population. The screening recommendations by the National Comprehensive Cancer Network apply to breast, thyroid, colon, endometrial, renal, and melanoma cancers [10,26].

## Conclusions

In patients with radiological and clinical suspicion of LDD and CS, it should be mandatory to investigate the presence of other types of tumors due to their association with PTEN hamartomatous tumor syndrome, and in the absence of genetic study, the clinical criteria previously established in the literature should be enough to establish the diagnosis. The foregoing provides an adequate diagnostic and management approach for the wide spectrum of pathologies that can be associated with LDD.
